# Neurological soft signs and structural network changes: a longitudinal analysis in first-episode schizophrenia

**DOI:** 10.1186/s12888-023-04522-4

**Published:** 2023-01-09

**Authors:** Li Kong, Christina J. Herold, Silke Bachmann, Johannes Schroeder

**Affiliations:** 1 Department of Psychology, Shanghai Normal University, 100 Guilin Road, Shanghai, China; 2 Section of Geriatric Psychiatry, Department of Psychiatry, University of Heidelberg, Heidelberg, Germany; 3 Department of Psychiatry, University of Genova, Geneva, Switzerland; 4 Department of Psychiatry, Martin Luther University of Halle-Wittenberg, Halle, Germany

**Keywords:** Neurological soft signs, Schizophrenia, Network, Longitudinal analysis

## Abstract

**Background:**

Neurological soft signs (NSS) are often reported in patients with schizophrenia and may vary with psychopathological symptoms during the course of disease. Many cross-sectional neuroimaging studies have shown that NSS are associated with disturbed network connectivity in schizophrenia. However, it remains unclear how these associations change over time during the course of disorder.

**Methods:**

In present study, 20 patients with first-episode schizophrenia and 20 controls underwent baseline structural magnetic resonance imaging (MRI) scan and at one-year follow-up. Structural network characteristics of patients and controls were analyzed using graph theoretical approach based on MRI data. NSS were assessed using the Heidelberg scale.

**Results:**

At baseline, patients demonstrated significant changes of the local network properties mainly involving regions of the cortical-subcortical-cerebellar circuits compared to healthy controls. For further analysis, the whole patient group was dichotomized into a NSS-persisting and NSS-decreasing subgroup. After one-year follow-up, the NSS-persisting subgroup showed decreased betweenness in right inferior opercular frontal cortex, left superior medial frontal cortex, left superior temporal cortex, right putamen and cerebellum vermis and increased betweenness in right lingual cortex. However, the NSS-decreasing subgroup exhibited only localized changes in right middle temporal cortex, right insula and right fusiform with decreased betweenness, and in left lingual cortex with increased betweenness.

**Conclusions:**

These findings provide evidence for brain network reorganization subsequent to clinical disease manifestation in patients with first-episode schizophrenia, and support the hypothesis that persisting NSS refer to progressive brain network abnormalities in patients with schizophrenia. Therefore, NSS could help to establish a better prognosis in first-episode schizophrenia patients.

**Supplementary Information:**

The online version contains supplementary material available at 10.1186/s12888-023-04522-4.

## Background

Neurological soft signs refer to subtle neurological abnormalities of motor and sensory functions and are often reported in patients with schizophrenia. NSS may vary with psychopathological symptoms during the course of the disease [1–5]. These changes of NSS along the course of schizophrenia were considered to reflect the progression of neurological abnormalities [1, 5]. Many prior studies showed that the severity of NSS is associated with morphometric abnormalities in multiple brain regions, such as decreased grey matter volume or cortical thickness in prefrontal cortex, superior and middle temporal cortex, pre- and post-central cortices, insula, cerebellum, basal ganglia and thalamus [6–9]. Moreover, NSS are also associated with abnormal functional activation in prefrontal, pre- and post-central cortices, and insula in patients with schizophrenia [9–12]. Especially, longitudinal studies from our group revealed that the severity of NSS varied with the clinical course [13] and referred to progressive grey matter morphometric changes during the course of disease in patients with first-episode schizophrenia, thus indicating that NSS might help to establish the prognosis in patients with schizophrenia [14].

More recently, network-based approaches provide a powerful access to brain topological organization complementary to conventional morphometric and functional measurements on a more global level [15, 16]. Several groups have examined the neural basis of NSS at network level in patients with schizophrenia [17–19]. In a recent study our group reported that the severity of NSS is associated with alterations of structural brain networks corresponding to the cortical-subcortical-cerebellar circuit in patients with schizophrenia [19]. Other studies also found NSS abnormalities to be correlated with altered functional connectivity in multiple brain networks in patients with schizophrenia [17, 18]. However, longitudinal studies investigating the associations between NSS and changes of network characteristics over time in patients with schizophrenia are rather scarce, although this approach reduces the potentially confounding effects of intra-individual variability.

In the present study, graph theory-based approaches were used to examine structural network characteristics and their associations with the severity of NSS. Graph theory is a powerful method for quantifying the brain as a complex network [20]. It provides unique insight into the structural architecture of the brain to assess the integration and segregation of the brain network. Therefore, based on our earlier findings of NSS related longitudinal grey matter morphometric changes and cross-sectional structural networks alterations, we extended our previous work by exploring the longitudinal relationship between the severity of NSS and changes of brain network characteristics during a one-year follow-up in patients with first-episode schizophrenia. We hypothesized that persisting NSS refer to progressive alterations of brain network characteristics in patients with first-episode schizophrenia.

## Methods and materials

### Participants

For the present study, we used data from a previous publication, in which we examined the longitudinal relationships between NSS and grey matter volume changes in first-episode schizophrenia [14]. Detailed information on the subjects’ characteristics has been given before [14]. They are exhibited in Table 1. Twenty participants (7 males) with first-episode schizophrenia were recruited from the Department of Psychiatry, University of Heidelberg, Germany. Patients’ diagnoses were established using the German version of the Structured Clinical Interview for the Diagnostic and Statistical Manual of Mental Disorders (DSM-IV) [21]. Psychopathological symptoms were rated on the Positive and Negative Syndrome Scale (PANSS) [22]. None of the patients had a history of severe substance abuse or neurological disorder. All patients were again examined after a mean duration of 13.8 months (Standard Deviation, SD = 1.6). At baseline patients received atypical antipsychotics with a mean dose of 549.3 mg/day (SD = 271.3) of chlorpromazine equivalents (CPZ; [23]). Sixteen patients (80%) adhered to psychiatric treatment regularly with a mean dose of 443.8 mg/day CPZ (SD = 137.7) during the follow-up period. Twenty healthy individuals were recruited through advertisements and included in the study. The group comprised 10 males and 10 females with a mean age of 24.1 years (SD = 3.5) and 11.9 years (SD = 1.5) of education. None of the participants had any major psychotic disorders, history of neurological or medical illness, head injury or substance abuse. The study was approved by the ethics committee of the Medical Faculty, University of Heidelberg, Germany. All participants provided written informed consent after full explanation of the proceedings.

**Table 1:** Clinical and demographic variables

Variables Mean (SD)		Healthy Controls	Patients (Baseline)	Patients (Follow-up)		*P*-value
Age (year)		24.1 (3.5)	25.6 (7.2)	/		0.52
Sex(male/female)		10/10	7/13	/		0.34
Chlorpromazine equivalents (mg/day)		/	549.3 (271.3)	443.8(137.7)		0.17
NSS		3.6 (1.6)	15.3 (6.8)	11.30 (8.31)		< 0.001^a^
NSS subgroups						
	Decreasing NSS (*n* = 10)			Persisting NSS (*n* = 10)		
	Baseline	Follow-up		Baseline	Follow-up	
NSS	15.9 (3.9)	7.2 (3.5)		14.6 (9.2)	14 (9.5)	< 0.001^b^
PANSS	51.2 (9.8)	44.2 (9.9)		50.1(13.6)	52.8(22.8)	0.28^b^
Age at baseline	26.1 (6.5)			25.0 (8.2)		0.66
Sex(male/female)	3/7			4/6		0.64

*NSS* Neurological soft signs, *PANSS* Positive and negative syndrome scale, *SD* Standard deviation

a: *p*-value for the difference between healthy controls and patients at baseline

b: repeated ANOVAs in two subgroups

NSS were assessed at baseline and follow-up with the Heidelberg Scale [4], which is well established and widely used in neuropsychiatric diseases [24–28]. It includes 5 subscales comprising 16 items. Ratings were given on a 0–3-point scale (no/slight/moderate/marked abnormality). In order to further investigate the changes of NSS over time, the whole patients group (20 patients) were divided into 2 subgroups based on the mean change of NSS total scores. There were 10 patients with a notable decrease in total NSS scores (NSS-decreasing subgroup: 3 males, 7 females; mean age: 26.1 years (SD = 6.5); change of NSS: 5 ~ 18) and 10 patients with persisting or increasing NSS level (NSS-persisting subgroup: 4 males, 6 females; mean age: 25.0 years (SD = 8.2); change of NSS: − 10 ~ 4). The changes of PANSS scores in both subgroups were also summarized in Table 1.

### MRI acquisition

Structural MRI data of all participants were obtained at the German Cancer Research Centre with a 1.5 T Magnetom Vision MR scanner (Siemens Medical Solutions, Erlangen, Germany) with the following parameters: magnetization-prepared rapid gradient echo (MP-RAGE), 126 coronal slices, image matrix = 256 × 256, voxel size = 0.98 × 0.98 × 1.8 mm^3^, repetition time (TR) = 10 ms, echo time (TE) = 4 ms, flip angle = 12°.

### Image preprocessing and analysis

In order to compare the present network results with our earlier grey matter volume findings from the same sample [14], we kept using the same preprocessing tools with VBM8 (http://dbm.neuro.uni-jena.de/vbm) in SPM8 (http://www.fil.ion.ucl.ac.uk/spm) on Matlab 2013 platform (http://www.mathworks.com/products/matlab). Detailed steps of VBM8 have been described in previous studies (Ashburner and Friston, 2000; Good et al., 2001). Briefly, it included (1) normalization of all images to a Montreal Neurological Institute (MNI) template; (2) normalized brain images were segmented into grey matter, white matter, and cerebrospinal fluid compartments; and (3) normalized grey matter images were modulated.

The WFU PickAtlas Toolbox was used to extract 116 regions of interest (ROIs) using the Automated Anatomical Labeling (AAL) atlas [29]. These cerebral ROIs were re-sliced based on the normalized grey matter images. The REX toolbox (https://web.mit.edu/swg/software.htm) was then used to extract ROI volumes from the modulated and normalized grey matter images.

### Network construction and measurements

In the present study, the 116 extracted AAL ROIs were defined as nodes, and the Pearson correlation coefficients between ROIs represented the edges. Structural correlation matrices were then constructed by calculating the correlation coefficients between regions for each group. Age and gender were entered as covariates to control for potential confounding effects. Binary adjacency matrices were then derived based on the association matrices by thresholding a range of densities. The lower bound of the range was determined in which all nodes were fully connected. The upper bound of the range was 0.5 according to previous results, as grey matter structural networks became less biological above this bound [30, 31].

To investigate the global topological properties of a network, clustering coefficient (C) and characteristic path length (L) were calculated [31–33]. The clustering coefficient of a node is a measurement of the number of connections between its directly neighboring regions, and the clustering coefficient of a network is the average of the clustering coefficient, which reflects the segregation of the network [31]. The characteristic path length is the average shortest passing through the node, which reflects the integration of the network. The small-world index of the network was defined as (C/C_rand_)/ (L/L_rand_), where C_rand_ and L_rand_ are the mean clustering coefficient and the characteristic path length of the random network [34]. A network with small-world index means that it has a significantly higher clustering coefficient than in random networks, and comparable characteristics path length to that in random networks [33].

Regional network characteristics of the constructed structural networks were assessed based on nodal betweenness centrality for each of the anatomical ROIs at minimum density with full connectivity for the network. Nodal betweenness represents the fraction of all shortest paths in the network that pass through, which can defect important anatomical or functional connections [35].

The hubs represent the importance of the nodes and are crucial regulators for efficient information communication in brain networks [31]. A node was set as a hub if its betweenness was at least two SDs above the mean betweenness [33, 36].

### Statistical analysis

The clinical and demographic data were analyzed using Student’s t tests for continuous variables and Chi-squared tests for categorical variables in SPSS 22. Repeated measures ANOVA were performed to examine time effects and group effects for NSS and PANSS scores.

To investigate the differences of the global and regional network measures between groups, parametric t-tests and nonparametric tests with 1000 repetitions were performed using GAT software [33, 36]. After calculating group differences at each density, areas under curve (AUC) were then performed for each network measures so that the between-group differences were less sensitive to the thresholding values. In an exploratory fashion, we assessed changes in network characteristics over one-year follow-up between the two subgroups. Two-tailed p values were obtained based on its percentile position [33, 37], and false discovery rate (FDR) corrected p values were also reported to correct for multiple comparisons with *p* < 0.05.

## Results

### Demographic data

The clinical and demographic characteristics of the sample are summarized in Table 1. There were no significant differences in age and sex between healthy controls and patients with schizophrenia. NSS scores in patients are significantly higher than in healthy controls (*p* < 0.001). Repeated measures ANOVA analysis showed a significant reduction of NSS in the decreasing-NSS subgroup compared to that in the persisting-NSS subgroup (*p* < 0.001). In addition, compared with the persisting-NSS subgroup, the decreasing-NSS subgroup tended to have a more favorable one-year outcome as indicated by scores on the PANSS; however, the differences between the two subgroups were not statistically significant.

### Cross-sectional network measurements in the whole group

There were no significant differences with respect to global network measures between patients with schizophrenia and healthy controls at baseline. AUC analysis showed that patients with schizophrenia demonstrated significant changes in local network properties and network hubs compared to controls mainly involving regions of cortical-subcortical-cerebellar circuits. Detailed information is described in the supplementary file (Figure S1 for local network properties and Figure S2 for network hubs).

### Longitudinal network measurements in two subgroups

Both NSS-persisting and NSS-decreasing subgroups followed small-world characteristics at baseline and follow-up. After the one-year follow-up, both subgroups did not show significant global network differences including the small-world index, clustering coefficient and path length.

**Fig. 1:**
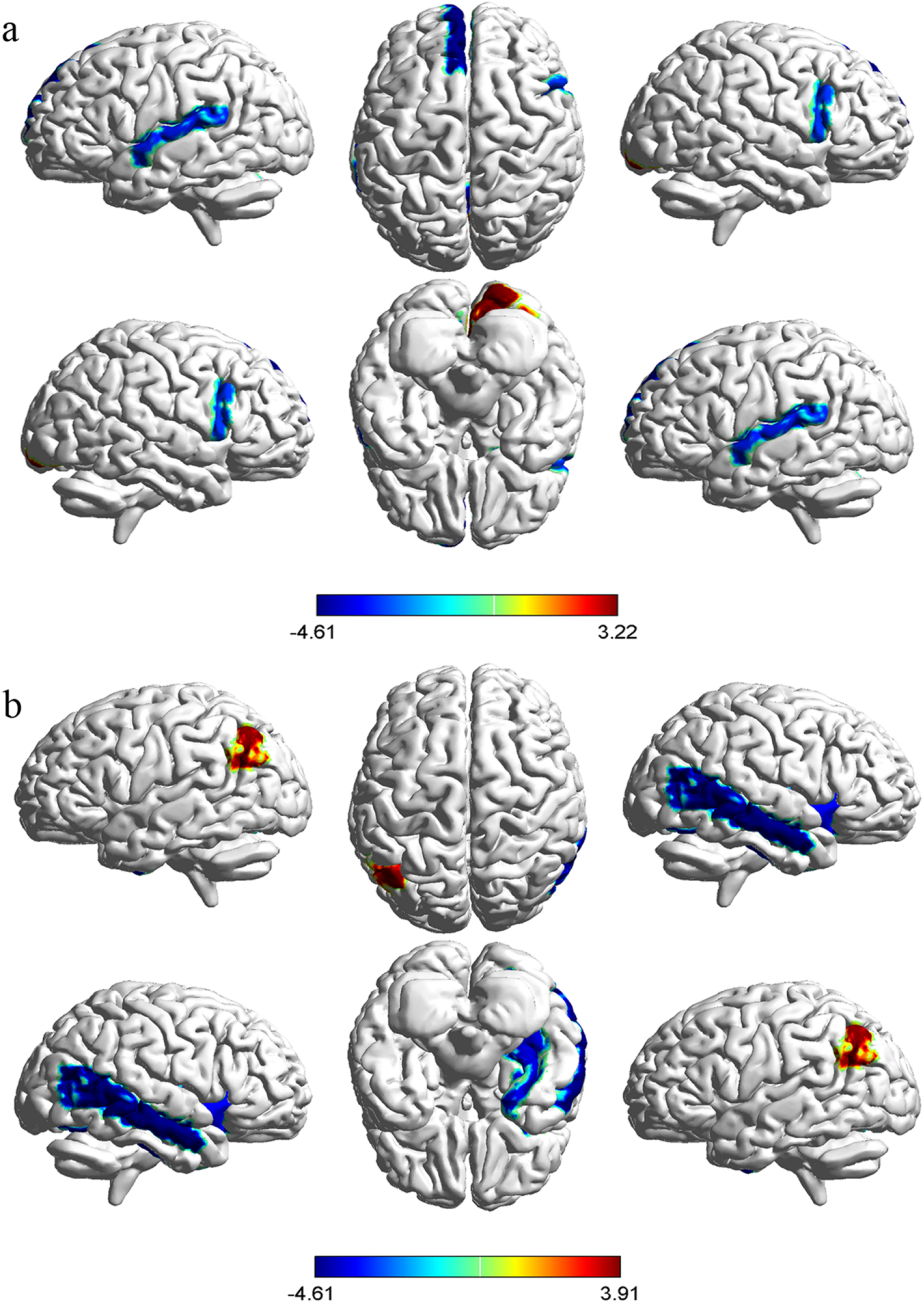
The betweenness changes in NSS-persisting (**a**) and NSS-decreasing (**b**) subgroups from baseline to 1-year follow-up; Hot color represents significantly increased nodal betweenness and cold color represents significantly decreased nodal betweenness. The results were obtained from AUC analysis

AUC analysis showed that the NSS-persisting subgroup developed longitudinally significant reductions of local betweenness mainly involving right opercular inferior frontal cortex, left medial superior frontal cortex, left superior temporal cortex, right putamen and cerebellum vermis, while betweenness became larger in right lingual cortex (Fig. 1a). For the NSS-decreasing subgroup, patients demonstrated a significant smaller betweenness at follow-up in right middle temporal cortex, right insula and right fusiform, while betweenness became larger in left lingual cortex. However, none of the involved regions survived the correction for multiple comparisons (*p* < 0.05, FDR corrected) (Fig. 1b).

**Fig. 2:**
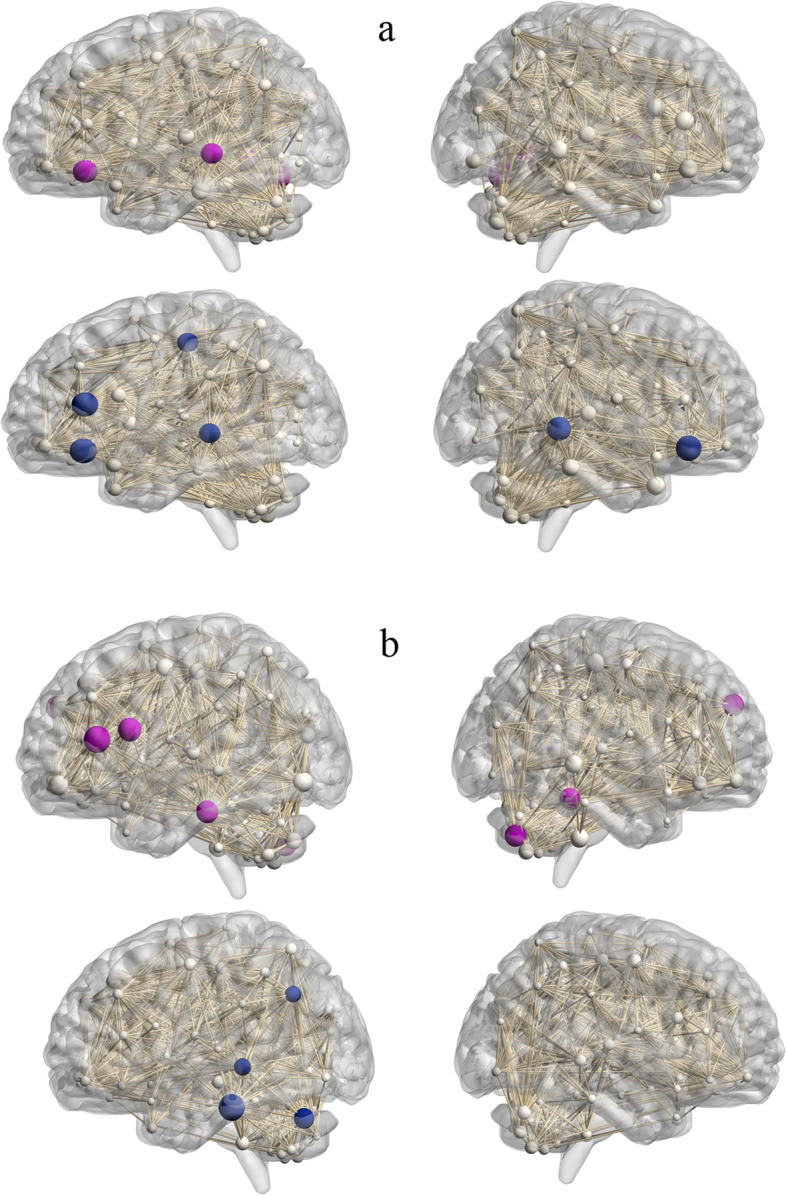
Network hubs for NSS-persisting subgroup (**a**) and NSS-decreasing subgroup (**b**). Network hubs were labeled (2SD larger than mean betweenness). The size of the sphere means the betweenness of the corresponding region. Purple and blue colors represent hubs for two subgroups at baseline and one-year follow-up, respectively. The results were obtained from AUC analysis

The network hubs of the NSS-persisting subgroup at baseline were identified in left orbital inferior frontal cortex, left middle temporal cortex, right putamen, and cerebellum vermis; after one-year follow-up, the network hubs were observed in left and right orbital inferior frontal cortices, left triangular inferior frontal cortex, left postcentral cortex and left and right middle temporal cortices (Fig. 2a). The network hubs of the NSS-decreasing subgroup at baseline were identified in left opercular and triangular inferior frontal cortices, right medial superior frontal cortex, left inferior temporal cortex, right fusiform and right cerebellum; after one-year of follow-up, the network hubs located in left middle and inferior temporal cortices, left angular and left cerebellum (Fig. 2b).

## Discussion

In the present study, we conducted a longitudinal design to link NSS trajectories with dynamic changes in structural network properties during the course of first-episode schizophrenia. The main results were as follows: (1) the NSS-persisting subgroup showed more widespread local betweenness changes at follow-up involving frontal and temporal cortices, insula, putamen and cerebellum than the NSS-decreasing subgroup; (2) different alterations of network-hub distributions were observed between NSS-persisting and NSS-decreasing subgroups from baseline to follow-up.

Before splitting the whole group into NSS-persisting and NSS-decreasing subgroups, we first performed a cross-sectional analysis to compare the network properties between patients with schizophrenia and healthy controls. Our results showed that there were no significant differences in global network measures between patients with schizophrenia and healthy controls. However, regional network analyses showed significantly changed betweenness centrality, especially in areas of cortical-subcortical-cerebellar circuits. These results were generally consistent with previous cross-sectional studies investigating structural network characteristics between patients with schizophrenia and healthy controls, and further confirmed a disturbed cortical-subcortical-cerebellar circuitry in schizophrenia [38–40].

Further longitudinal analyses demonstrated that both NSS-persisting and NSS-decreasing subgroups did not demonstrate significant changes in global network topologies over one-year follow-up. This stability suggests that crucial characteristics of the network are highly preserved over one-year follow-up in patients with first-episode schizophrenia. With respect to the local betweenness centrality, the NSS-persisting subgroup showed a more widespread variety in betweenness centrality than the NSS-decreasing subgroup at follow-up in contrast to baseline, mainly involving right opercular inferior frontal cortex, left medial superior frontal cortex, left superior temporal cortex, right putamen and cerebellum vermis. The present results not only provide additional evidence that brain network topologies change over time, but also suggest that NSS severity was associated with progressions of network characteristics, especially involving the cortical-subcortical-cerebellar circuit in patients with fist-episode schizophrenia. This NSS-related brain network variety over time is generally in line with results from our previous cross-sectional study that showed NSS severity to be associated with alterations in topological attributes of brain networks corresponding to the cortical-subcortical-cerebellar circuit in patients with schizophrenia [19]. Alterations in local network properties in these regions indicated an abnormal ability for modulating information flow and participating in functional interactions with their adjacent regions [33]. Therefore, NSS may present a clinical phenotype of the dynamic changes in brain networks that putatively underlies the neurobiology of schizophrenia.

In addition, in our previous longitudinal study in which we investigated NSS-related grey matter morphometric changes over one-year follow-up, we reported more pronounced grey matter volumetric reduction over time in the NSS-persisting group than in the NSS-decreasing group mainly located in cortical structures and cerebellum [14]. A significant difference to the present results is that we now identified NSS-related network variety over time, which also includes subcortical putamen in addition to cortical and cerebellar regions. Prior cross-sectional studies investigating NSS-related grey matter morphometric characteristics often described that NSS are associated with abnormalities in the putamen [6, 41]. The putamen receives input from sensorimotor cortex and related to other parts of the basal ganglia and cortical structures, which plays an important role in modulating sequential motor functions [42]. Some studies also reported that smaller putamen is associated with poorer outcome in schizophrenia [43]. However, our earlier longitudinal study based on morphometric analysis could not identify significant changes of putamen volume during disease duration of one year [14]. Instead, the present network analysis revealed quantifiable varieties in putamen during the one-year follow-up period, which may suggest that network analysis is much more sensitive to minor changes in rather small cerebral structures than morphometric methods.

Network analysis further identified alterations of hub distribution in both subgroups from baseline to one-year follow-up. The network hubs of the NSS-persisting subgroup changed significantly over time mainly involving inferior frontal cortex, middle temporal cortex, postcentral cortex, putamen and cerebellum. The network hubs of the NSS-decreasing subgroup changed significantly over time mainly involving inferior frontal cortex, inferior and middle temporal cortices, angular and cerebellum. Similar to the above-described results of local betweenness, the alterations of hubs in the subcortical region putamen were only identified in the NSS-persisting subgroup, especially at the baseline assessment. These results suggest that the abnormality of the putamen at baseline may relate to the persisting of NSS one year later and further heralds chronicity of schizophrenia. Hubs are key parts of efficient information communication and regulation in a network [33]. The present NSS-related alterations of hub distributions indicate a less efficient information transmission in the cortical-subcortical-cerebellar circuitry and also suggest a reconfiguration of brain networks. These results further confirm that NSS are related to disturbed cortical-subcortical-cerebellar circuitry in schizophrenia longitudinally at network level.

In addition, our results also indicate that the decreasing-NSS subgroup tend to have a more favorable outcome with respect to PANSS sum score compared to the persisting-NSS subgroup, although the difference did not reach significance. However, whether there is a parallel development between psychopathology (PANSS) and NSS in schizophrenia remains unclear. Some studies demonstrated a parallelism of PANSS with the severity of NSS in schizophrenia [44], but others not [45]. Nevertheless, our present findings indicate that grouping patients based on NSS could create more homogeneous subgroups, therefore allowing a more sensitive detection of network changes linked to the course of schizophrenia.

We have also shown that distinct patterns of networks correlated significantly with NSS severity over time in patients with schizophrenia. The utilization of longitudinal network-NSS profiling analysis allowed us to tighter differentiate between clinical phenotypes (i.e. NSS-groups) and their underlying neurobiology, thus providing important information to complement existing methods of patient assessment.

The main strength of the present study is its longitudinal design to investigate NSS-related structural changes at a network level in schizophrenia. A potential limitation of the study is our small sample size, which may limit the generalization of our results. Additionally, the lack of a healthy control group at follow-up limits our ability to comment on whether the patterns of NSS related network changes are different from those in healthy participants. However, a recent study investigating network changes in healthy controls over one year did not identify significant variations of network properties [46]. Effects of medication are also potential confounding variables, although clinical studies demonstrated that NSS are not the sequelae of antipsychotic treatment [47].

## Conclusions

To the best of our knowledge, this is the first longitudinal study that focused on to NSS trajectories with dynamic changes in structural network properties in patients with schizophrenia. Our results suggest that NSS severity is associated with variability of structural network characteristics, especially involving cortical-subcortical-cerebellar circuits, which may underlie the heterogeneity of NSS trajectories in first-episode schizophrenia. The present results may provide a new perspective for elucidating the neural basis of NSS in schizophrenia.

## Supplementary Information

**Additional file 1:** **Figure S1.** Group differences of patients with schizophrenia and healthy controls. Significantly increased (hot color) and reduced (cold color) nodal betweenness were observed in patients with schizophrenia. The results were obtained from AUC analysis. **Figure S2.** Network hubs for patients with schizophrenia and healthy controls. Network hubs were labeled (2SD larger than the mean betweenness).

## Data Availability

The data are available from the corresponding author upon reasonable request.

## Abbreviations

NSS: Neurological soft signs
MRI: Magnetic resonance imaging
DSM-IV: Diagnostic and statistical manual of mental disorders
SD: Standard deviation
CPZ: Chlorpromazine equivalents
MP-RAGE: Magnetization-prepared rapid gradient echo
TR: Repetition time
TE: Echo time
MNI: Montreal neurological institute
AAL: Automated anatomical labeling
ROIs: Regions of interest
AUC: Areas under curve
FDR: False discovery rate
PANSS: Positive and Negative Syndrome Scale
