# Supplementary material for: Neurological soft signs and structural network changes: a longitudinal analysis in first-episode schizophrenia
Source: BMC Psychiatry. 2023 Jan 9;23:20. doi: 10.1186/s12888-023-04522-4 (PMC9830771; doi:10.1186/s12888-023-04522-4)
Supplement: Supplementary file 1 — Additional file 1: Figure S1. Group differences of patients with schizophrenia and healthy controls. Significantly increased (hot color) and reduced (cold color) nodal betweenness were observed in patients with schizophrenia. The results were obtained from AUC analysis. Figure S2. Network hubs for patients with schizophrenia and healthy controls. Network hubs were labeled (2SD larger than the mean betweenness). [file 12888_2023_4522_MOESM1_ESM.docx]

**Supplementary Materials for**

**Neurological soft signs and structural network changes: A longitudinal analysis in first-episode schizophrenia**

There were no significant differences in global network measures including clustering coefficient (p=0.84), characteristic path length (p=0.27) and small-world index (p=0.73) between patients with schizophrenia and healthy controls at baseline.

AUC analysis showed that compared with healthy controls at baseline, patients demonstrated smaller betweenness mainly involving right orbital superior frontal cortex, right inferior temporal cortex, right parahippocampal cortex, right inferior occipital cortex, left superior parietal cortex and right cerebellum, while betweenness was larger in left medial superior frontal cortex, right superior frontal cortex, left fusiform, left middle occipital cortex, bilateral caudate, right putamen and cerebellum (Figure S1). However, only differences in right orbital superior frontal cortex and right inferior occipital cortex remained significant after correction for multiple comparisons (p<0.05, FDR corrected).

Network hubs of the healthy controls based on nodal betweenness were identified in right amygdala, right orbital superior frontal cortex, right middle and inferior temporal cortices, left superior parietal cortex and left cerebellum. In patients with schizophrenia at baseline network hubs were observed in right caudate, left medial superior frontal cortex, right middle temporal cortex, left postcentral cortex, left fusiform, left middle occipital cortex and right cerebellum (Figure S2).


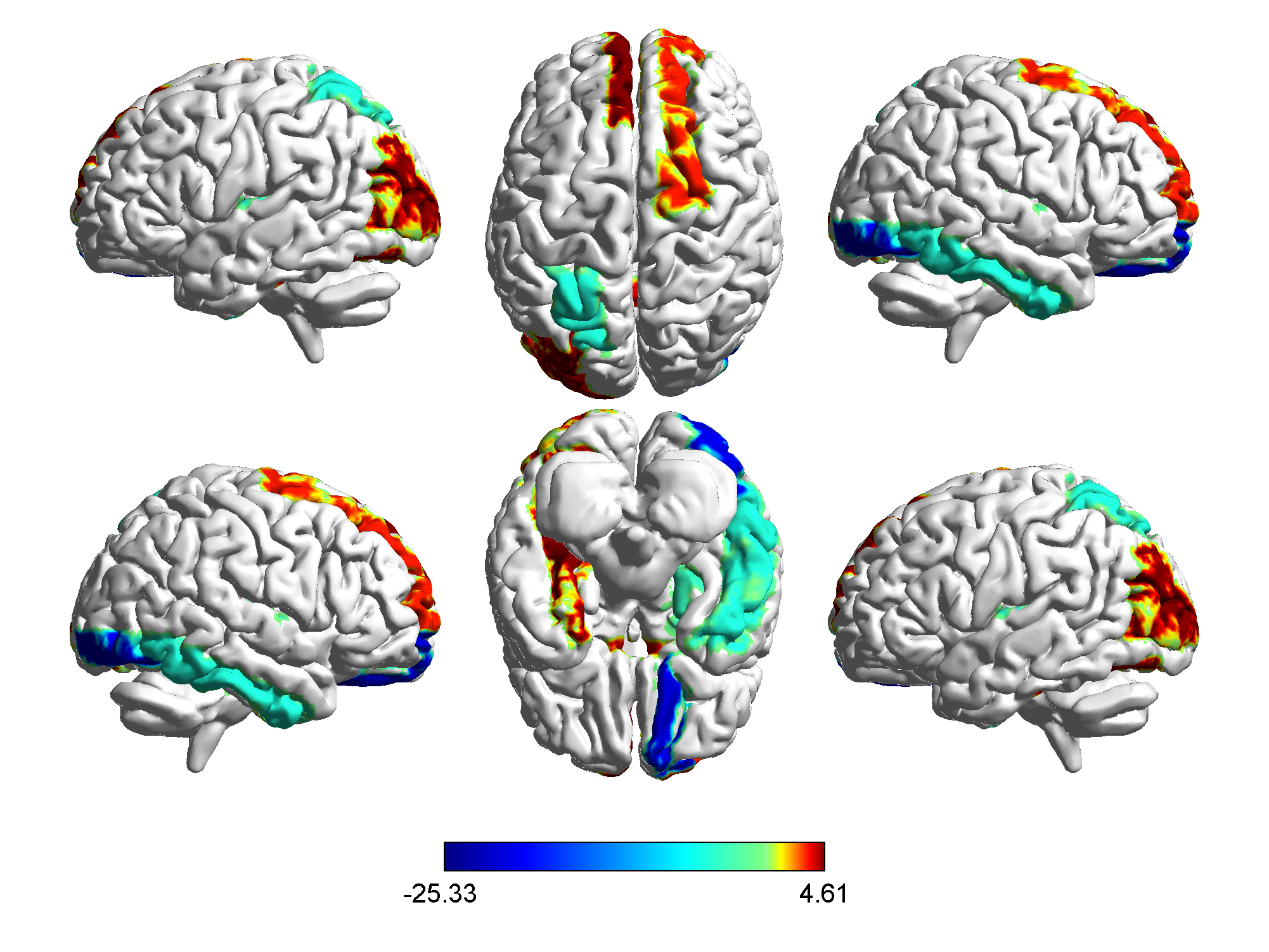


Figure S1 Group differences of patients with schizophrenia and healthy controls. Significantly increased (hot color) and reduced (cold color) nodal betweenness were observed in patients with schizophrenia. The results were obtained from AUC analysis.


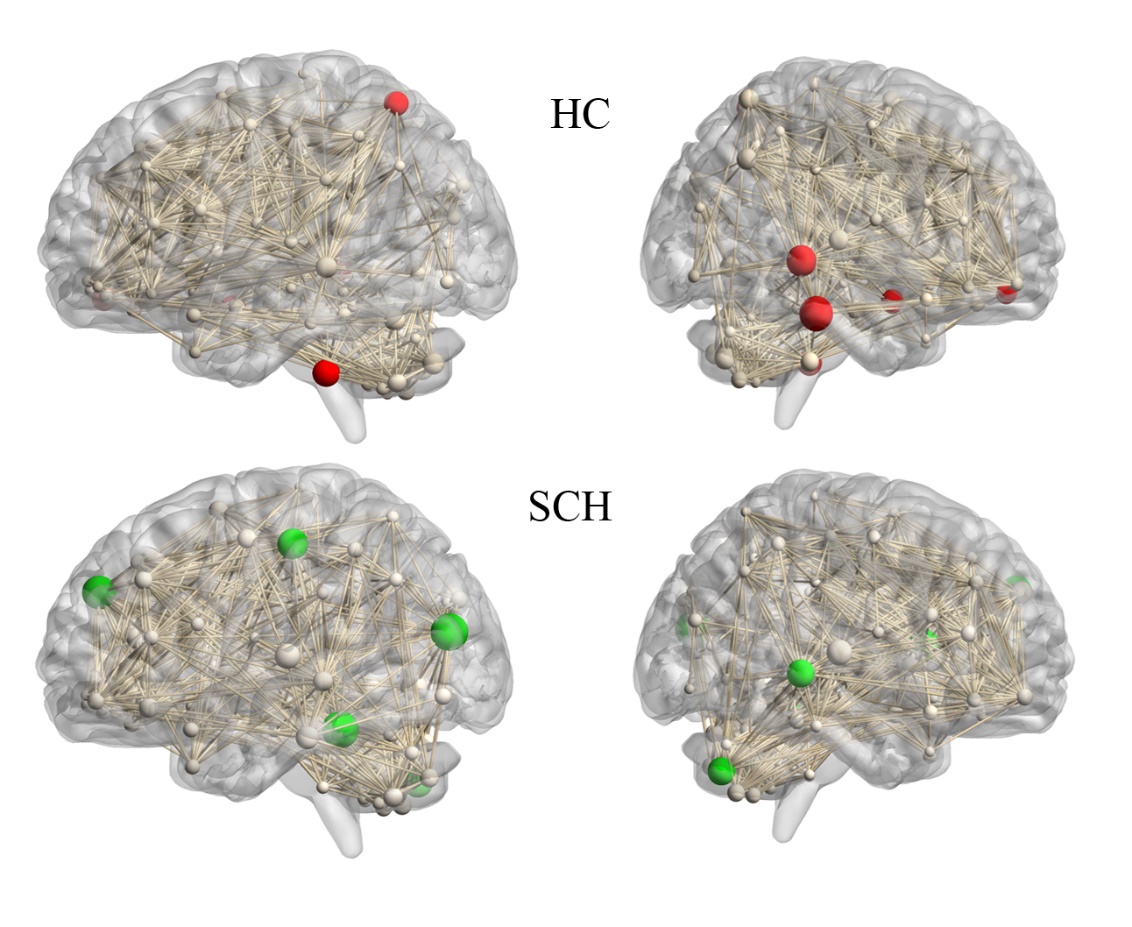


Figure S2 Network hubs for patients with schizophrenia and healthy controls. Network hubs were labeled (2SD larger than the mean betweenness). The size of the sphere means the betweenness of the corresponding region. Red color refers to the healthy controls. Green color refers to patients with schizophrenia. The results were obtained from AUC analysis. HC: Healthy controls; SCH: patients with schizophrenia
